# Treatment Outcomes of HER2-Directed Therapy in Patients With HER2-Positive Non-metastatic Breast Cancer in Low-Resource Settings

**DOI:** 10.7759/cureus.90634

**Published:** 2025-08-20

**Authors:** Maryam Imran, Muhammad Awais Majeed, Sameen Bin Naeem, Muhammad Ahsan Jamil, Minahil Shahzad, Fatima Akhtar, Amna Karim, Aftab Ahmad, Shehar Bano, Beenish Aqib, Syed Abdullah Javaid Bukhari

**Affiliations:** 1 Medical Oncology, Shaukat Khanum Memorial Cancer Hospital and Research Centre, Lahore, PAK; 2 Oncology, Shaukat Khanum Memorial Cancer Hospital and Research Centre, Lahore, PAK; 3 Medical Officer, Shaukat Khanum Memorial Cancer Hospital and Research Centre, Lahore, PAK

**Keywords:** her2-directed therapy, non-metastatic breast cancer, pathological complete response, pertuzumab, residual disease, trastuzumab

## Abstract

Introduction

This study aimed to evaluate treatment outcomes of human epidermal growth factor receptor 2 (HER2)-directed therapies in patients with non-metastatic HER2-positive breast cancer treated in a low-resource setting. Specifically, we assessed the impact of dual blockade (trastuzumab and pertuzumab), trastuzumab alone, or no HER2-targeted therapy on rates of residual disease, pathological complete response (pCR), progression-free survival (PFS), and overall survival (OS).

Methods

We conducted a retrospective cohort study at Shaukat Khanum Memorial Cancer Hospital, including 299 patients with non-metastatic HER2-positive breast cancer treated with neoadjuvant chemotherapy and either dual HER2 blockade, trastuzumab alone, or no HER2-targeted therapy due to financial constraints. Patient demographics, clinical features, treatments, and outcomes were analyzed using descriptive statistics, chi-square tests, and Kaplan-Meier survival analysis.

Results

The median age at diagnosis was 45.7 years (standard deviation±8.9). A majority of patients were premenopausal (n=222; 74.2%), and the majority presented with a palpable lump (n=275; 91.9%). Tumors were mainly located in the left (n=149; 49.8%) or right breast (n=147; 49.2%), with bilateral involvement in 3 (1.0%) cases. Invasive ductal carcinoma was the predominant histology (n=275; 91.9%), with estrogen receptor and progesterone receptor positivity observed in 185 (61.9%) and 179 (59.9%) patients, respectively. Grade III tumors were observed in 156 (52.2%) cases, and most tumors were T2 stage (n=236; 78.9%) with axillary nodal involvement in 232 (77.6%).

Patients receiving dual HER2 blockade achieved a pCR in 45 (54.9%) of 82 cases, compared to 51 (45.9%) of 111 with trastuzumab alone, and 39 (36.8%) of 106 with no HER2 therapy (p=0.046). The docetaxel, carboplatin, trastuzumab, and pertuzumab (TCHP) regimen had the highest pCR rate in 19 (65.5%) of 29 patients (p<0.001). Grade III tumors were associated with higher pCR than Grade II (n=96; 56.5% vs. n=39; 30.2%; p<0.001).

At 60 months, PFS was 236 (79.0%) overall, highest in the dual blockade group (n=73; 89.0%), followed by trastuzumab (n=96; 86.5%) and no HER2 therapy (n=69; 65.1%). OS at 60 months was 271 (90.6%), highest in the dual blockade group (n=78; 95.1%), then trastuzumab (n=102; 91.9%) and no HER2 therapy (n=79; 74.5%). Achieving pCR was associated with improved PFS and OS. Differences in both outcomes across groups were statistically significant (p<0.001).

Conclusion

Dual HER2 blockade significantly improved pCR, PFS, and OS in non-metastatic HER2-positive breast cancer. These findings support the inclusion of HER2-targeted agents in standard neoadjuvant treatment, even in resource-limited settings. Addressing barriers to access remains essential to improving global outcomes in breast cancer care.

## Introduction

Breast cancer (BC) is the second most common malignancy and a leading cause of morbidity and mortality among women globally [1]. According to Global Cancer Statistics, BC accounts for approximately 11.7% of all new cancer cases worldwide [2]. Among the various subtypes of BC, human epidermal growth factor receptor 2 (HER2)-positive BC is known for its aggressive behavior, comprising 15% to 20% of BC cases [3,4]. It is characterized by the overexpression of the HER2 receptor on the surface of cancer cells and necessitates specialized HER2-targeted therapies [3,5].

The introduction of HER2-targeted therapies, particularly trastuzumab and pertuzumab, has transformed BC management. These agents inhibit HER2 protein overexpression, thereby limiting cancer cell proliferation and improving treatment outcomes, progression-free survival (PFS), and overall survival (OS) rates [4,5]. This treatment paradigm has become standard care for HER2-positive BC in high-income countries [6,7]. Evidence from the NeoSphere trial showed that five-year PFS was 81% for patients receiving trastuzumab plus docetaxel, 86% for those given pertuzumab and trastuzumab plus docetaxel, and 73% for patients treated with either pertuzumab and trastuzumab or pertuzumab and docetaxel [5].

However, the feasibility and effectiveness of these therapies in resource-limited settings remain uncertain [8]. These targeted treatments are often cost-prohibitive and inaccessible to the majority of patients. The situation is exacerbated by the limited availability of diagnostic modalities necessary to confirm HER2 status, including immunohistochemistry, fluorescence in situ hybridization, and chromogenic in situ hybridization [8]. Consequently, many patients either do not receive the appropriate treatment or are diagnosed at advanced stages, thereby limiting treatment efficacy [9].

In Pakistan, over 148,000 new cancer cases are diagnosed annually, with approximately 100,000 cancer-related deaths [10]. Among these, BC is the most prevalent, with a frequency of 21.4% across both sexes and 38.8% in females [11]. Despite this high incidence, access to timely and adequate cancer care remains suboptimal. A Pakistani study reported that only 30%-40% of patients received standard chemotherapy for BC, and less than 10% of HER2-positive patients completed the recommended 17 cycles of HER2-targeted therapy within one year. Moreover, about 50% of patients experienced dose delays, regimen modifications, or discontinuation of therapy [9].

Given the challenges of implementing HER2-targeted therapies in low-resource environments, it is essential to evaluate their outcomes and identify feasible modifications. With recent advancements in BC management, these therapies are gradually becoming more affordable and accessible. This study aimed to evaluate the treatment outcomes, including pathological complete response (pCR) and disease-free survival, of HER2-directed therapies in patients with non-metastatic HER2-positive BC in resource-limited settings. The findings intend to contribute to ongoing efforts aimed at improving BC treatment outcomes in Pakistan.

## Materials and methods

A retrospective cohort study was conducted at the Shaukat Khanum Memorial Cancer Hospital and Research Centre from December 2018 to May 2020, following ethical approval from the Institutional Review Board (approval letter number: EX-21-03-24-01), which granted a waiver of informed consent. A total of 299 patients were enrolled. All patients in this study received chemotherapy (n=299), which included anthracyclines and taxanes. In contrast, some patients received single-agent trastuzumab along with chemotherapy (n=111), and some patients received trastuzumab and pertuzumab along with chemotherapy (n=82). Inclusion criteria comprised patients aged <65 years with HER2-positive non-metastatic BC confirmed via immunohistochemistry. Patients with metastatic disease or equivocal HER2 status were excluded.

Data were retrospectively extracted from medical records. Variables collected included demographic data (age, age at menarche, and menopausal status), clinical information (symptom duration, follow-up duration, presenting complaints, grade, tumor size, histopathological findings, estrogen receptor (ER) and progesterone receptor (PR) status, and nodal status), treatment details (neoadjuvant chemotherapy and HER2-directed therapy), and follow-up data (latest mammogram and survival status).

HER2 status was confirmed through immunohistochemistry. Histopathological and radiological records were reviewed to validate the diagnosis and disease stage. Data were entered into SPSS version 26 (IBM Corp., Armonk, NY) for analysis. Quality control measures included double-entry and cross-validation. Follow-up data were obtained from clinical records. Patients were categorized into three cohorts: those receiving both pertuzumab and trastuzumab, trastuzumab only, or no HER2-directed therapy.

Associations between categorical variables, such as HER2 therapy and surgical outcomes, were evaluated using chi-square tests. Survival analysis, including Kaplan-Meier curves and hazard functions, was performed to compare survival outcomes between groups (HER2-directed therapy single vs. dual blockade or no HER2-directed therapy).

pCR was defined as the absence of invasive cancer in the breast and axillary lymph nodes (ypT0N0 or ypTisN0) on final surgical pathology. PFS was defined as the time from initiation of first chemotherapy to radiological evidence of progression. OS was defined as the time from initiation of first chemotherapy to last follow-up or death.

ER and PR are considered positive if the result is more than 1% by immunohistochemistry.

## Results

A total of 299 patients were included in this study. The mean age was 45.7 years (standard deviation (SD)±8.9), and the mean age at menarche was 13.4 years (SD±0.9). The duration of symptoms ranged from 0.25 to 18 months. Among the patients, 222 (74.2%) were premenopausal, 72 (24.1%) were postmenopausal, and 5 (1.7%) had undergone surgical menopause.

Tumors were almost equally distributed between the left breast (149, 49.8%) and the right breast (147, 49.2%), with bilateral involvement observed in 3 (1.0%) cases. The most common presenting symptom was a palpable breast lump (275, 91.9%), followed by lump and nipple discharge (10, 3.3%), then lump and nipple retraction (9, 3.0%). This is shown below in Table 1.

**Table 1:** Baseline features of the study participants SD: Standard deviation

Variables	N (%) or mean ± SD
Age (years)	45.7±8.9
Age at menarche (years)	13.4±0.9
Duration of symptoms (months)	4.3±3.4
Duration of follow-up (months)	38.7±13.5
Menopausal status	–
Premenopausal	222 (74.2%)
Postmenopausal	72 (24.1%)
Surgical menopause	5 (1.7%)
Side involved	–
Left	149 (49.8%)
Right	147 (49.2%)
Bilateral	3 (1.0%)
Presenting complaints	–
Lump	275 (91.9%)
Discharge	1 (0.3%)
Lump + discharge	10 (3.3%)
Lump + retraction	9 (3.0%)
Multiple	4 (1.3%)

On histopathology, invasive ductal carcinoma was the predominant subtype, observed in 275 (91.9%) patients. ER positivity was observed in 185 (61.9%) patients and PR positivity in 179 (59.8%) patients. High-grade tumors (Grade III) were noted in 156 (52.2%) patients. Most tumors were classified as T2 (236, 78.9%), and nodal metastases were present in 232 (77.6%) of the cohort. This is shown below in Table 2.

**Table 2:** Patients’ diagnoses and clinical features DCIS: Ductal carcinoma in situ; T1: Tumor ≤2 cm; T2: Tumor >2 cm but ≤5 cm; T3: Tumor >5cm

Category	Subcategory	N	Percent
Histopathology	Invasive ductal carcinoma	275	91.9%
	Invasive ductal carcinoma + DCIS	24	8.0%
Estrogen receptor	Positive	185	61.9%
	Negative	114	38.1%
Progesterone receptor	Positive	179	59.8%
	Negative	120	40.1%
Grade	II	143	47.8%
	III	156	52.2%
Initial size	T1	21	7.0%
	T2	236	78.9%
	T3	42	14.0%
Nodal status	Positive	232	77.6%
	Negative	65	21.7%
	Not known	2	0.6%

Patients who received dual HER2 blockade with trastuzumab and pertuzumab achieved a pCR rate of 45 (54.9%), compared to 51 (45.9%) in those treated with trastuzumab alone and 39 (36.8%) among patients who did not receive any HER2-directed therapy. The overall chi-square test revealed a statistically significant difference in pCR rates among the three HER2 therapy groups (χ²=6.152, df=2, p=0.046). This is shown below in Table 3.

**Table 3:** Treatment and outcomes HER: Chemotherapy and trastuzumab; HER2: Human epidermal growth factor receptor 2; n: Number of patients; pCR: Pathological complete response; RD: Residual disease

HER2 therapy	pCR, n (%)	RD, n (%)	Total, n (%)	p-value vs. no HER2
No HER2	39 (36.8%)	67 (63.2%)	106 (35.5%)	–
Trastuzumab	51 (45.9%)	60 (54.1%)	111 (37.1%)	0.191
HER + pertuzumab	45 (54.9%)	37 (45.1%)	82 (27.4%)	0.014
Total	135 (45.2%)	164 (54.8%)	299 (100%)	-

The type of neoadjuvant chemotherapy regimen significantly influenced pCR outcomes. Patients treated with docetaxel, carboplatin, trastuzumab, and pertuzumab (TCHP) achieved the highest pCR rate (19, 65.5%), followed by those treated with doxorubicin, cyclophosphamide, paclitaxel, trastuzumab, and pertuzumab (AC-TAXOL-HERPER) (27, 49.1%), doxorubicin, cyclophosphamide, paclitaxel, and trastuzumab (AC-TAXOL-HER) (51, 45.5%), and doxorubicin, cyclophosphamide, and paclitaxel (AC-TAXOL) alone (38, 36.9%). The overall chi-square test revealed a statistically significant association between neoadjuvant chemotherapy regimen and pCR (χ²=8.345, df=3, p=0.039).This is shown below in Table 4.

**Table 4:** Outcomes based on the type of neoadjuvant treatment AC: Doxorubicin and cyclophosphamide; n: Number of patients; pCR: Pathological complete response; RD: Residual disease; Taxol: Paclitaxel; TCHP: Docetaxel, carboplatin, trastuzumab, and pertuzumab

Neoadjuvant therapy	pCR, n (%)	RD, n (%)	Total, n (%)	p-value vs. AC + Taxol
TCHP	19 (65.5%)	10 (34.5%)	29 (9.7%)	0.011
AC + Taxol	38 (36.9%)	65 (63.1%)	103 (34.4%)	–
AC + Taxol + trastuzumab	51 (45.5%)	61 (54.5%)	112 (37.5%)	0.251
AC + Taxol + trastuzumab + pertuzumab	27 (49.1%)	28 (50.9%)	55 (18.4%)	0.189
Total	135 (45.2%)	164 (54.8%)	299 (100%)	-

Patients who did not receive HER2 therapy, pCR was achieved in 10 (21.6%) patients with Grade II tumors and 29 (50.9%) patients with Grade III tumors (χ²=12.481, df=1, p<0.001). For those treated with chemotherapy and trastuzumab (HER), pCR was observed in 16 (34.8%) patients with Grade II tumors and 35 (53.8%) patients with Grade III tumors (χ²=3.942, df=1, p=0.047). In the group receiving trastuzumab and pertuzumab (HERPER), pCR occurred in 13 (40.6%) patients with Grade II tumors and 32 (64.0%) patients with Grade III tumors (χ²=4.305, df=1, p=0.038). Overall, Grade III tumors had a significantly higher pCR rate in 96 (56.5%) patients, compared to 39 (30.2%) patients with Grade II tumors (χ²=20.389, df=1, p<0.001). This is shown below in Table 5.

**Table 5:** Outcomes based on the grade HER2: Human epidermal growth factor receptor 2

HER2 therapy	Grade II pCR, n (%)	Grade III pCR, n (%)	Chi-square (χ²)	df	p-value
No HER2	10 (21.6%)	29 (50.9%)	12.481	1	<0.001
Chemotherapy + trastuzumab (HER)	16 (34.8%)	35 (53.8%)	3.942	1	0.047
Trastuzumab + pertuzumab (HERPER)	13 (40.6%)	32 (64.0%)	4.305	1	0.038
Overall	39 (30.2%)	96 (56.5%)	20.389	1	<0.001

**Figure 1:**
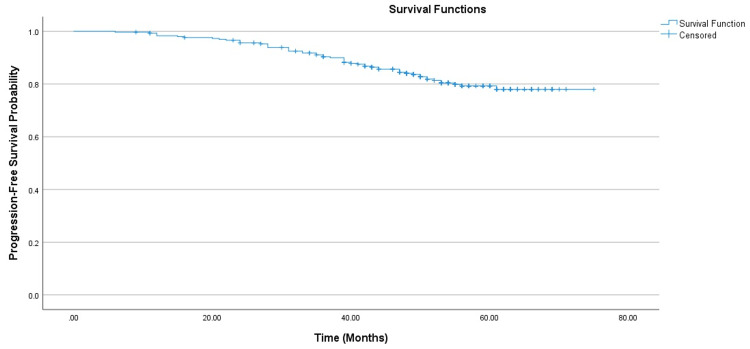
Kaplan-Meier curve demonstrating progression-free survival in patients with HER2-positive breast cancer. HER2: Human epidermal growth factor receptor 2

Long-term outcomes revealed that at 60 months, the overall PFS for the cohort was 79.0% (95% CI: 74.5%-84.4%), as shown in Figure 1.

**Figure 2:**
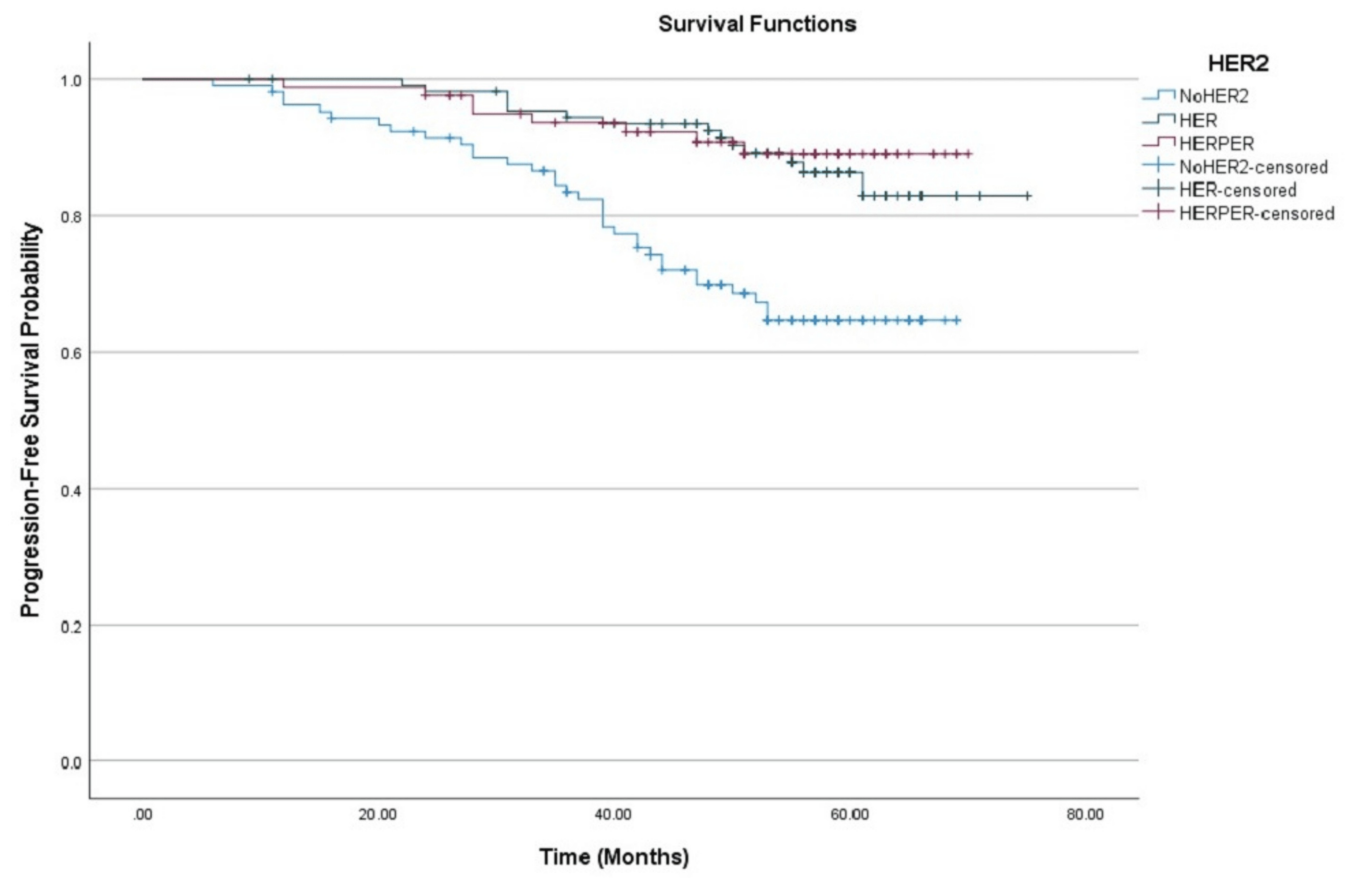
Kaplan-Meier curves showing progression-free survival among patients with HER2-positive breast cancer stratified by treatment group. HER2: Human epidermal growth factor receptor 2

Subgroup analysis showed marked differences in PFS based on HER2 treatment. Patients receiving dual HER2 blockade (trastuzumab + pertuzumab) demonstrated the highest PFS at 60 months (89.0%, 95% CI: 82.0%-96.6%), followed by those receiving trastuzumab alone (86.3% at 60 months, 95% CI: 79.5%-93.7%). The lowest PFS was observed in the group that did not receive HER2 therapy (64.5% at 60 months, 95% CI: 55.5%-75.1%), as shown in Figure 2.

**Figure 3:**
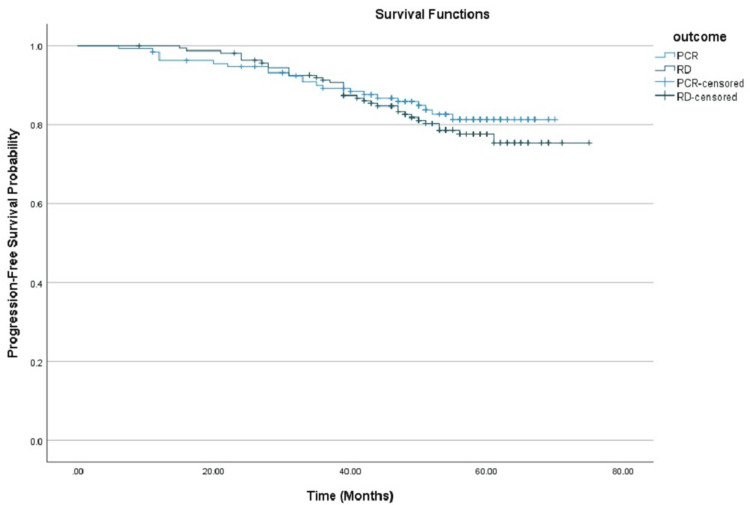
Kaplan-Meier curves comparing progression-free survival between patients who achieved pCR and those with RD. pCR: Pathological complete response; RD: Residual disease

Achieving pCR was also associated with superior PFS, with a 60-month rate of 81.3% (95% CI: 74.5%-88.9%), in contrast to 75.4% (95% CI: 67.9%-83.8%) in patients with residual disease, as shown in Figure 3.

**Figure 4:**
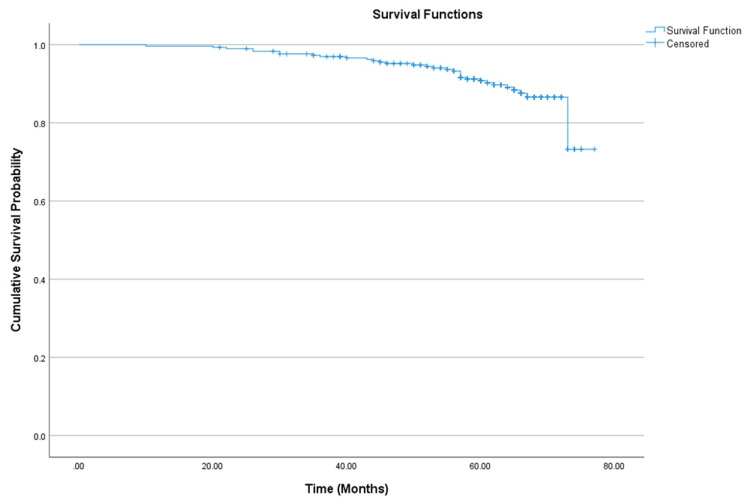
Kaplan-Meier curve demonstrating overall survival in patients with HER2-positive breast cancer. HER2: Human epidermal growth factor receptor 2

OS at 60 months was 90.8% (95% CI: 87.4%-94.3%), as shown in Figure 4.

**Figure 5:**
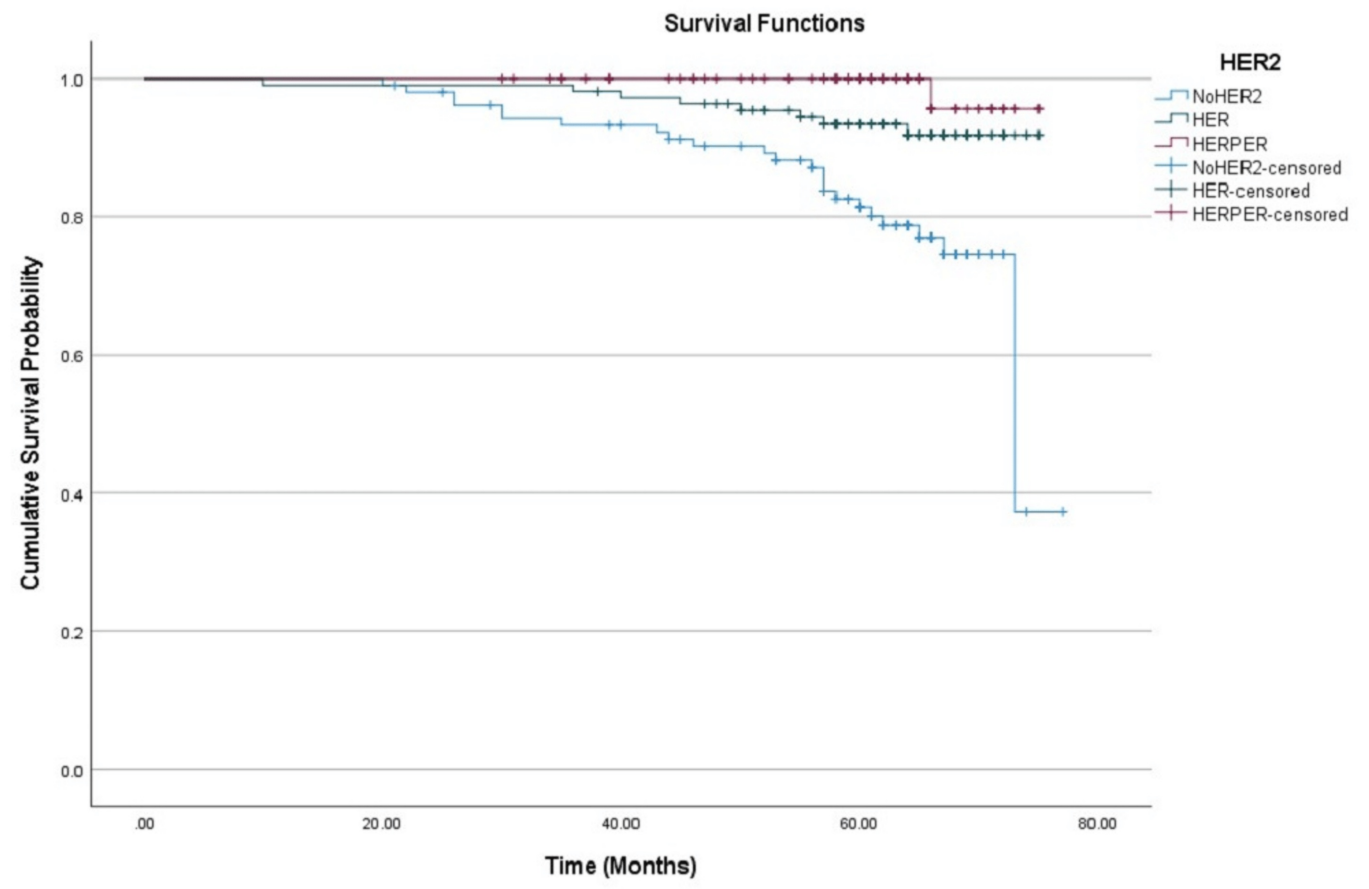
Kaplan-Meier survival curve comparing overall survival among patients with HER2-positive breast cancer stratified by treatment group. HER2: Human epidermal growth factor receptor 2

Dual HER2 blockade produced the most favorable OS (95.7% at 60 months, 95% CI: 87.7% to 100%), followed by trastuzumab alone (91.9%, 95% CI: 86.5%-97.6%), while patients without HER2 therapy had the lowest OS (74.6%, 95% CI: 65.5%-85.0%), as shown in Figure 5.

**Figure 6:**
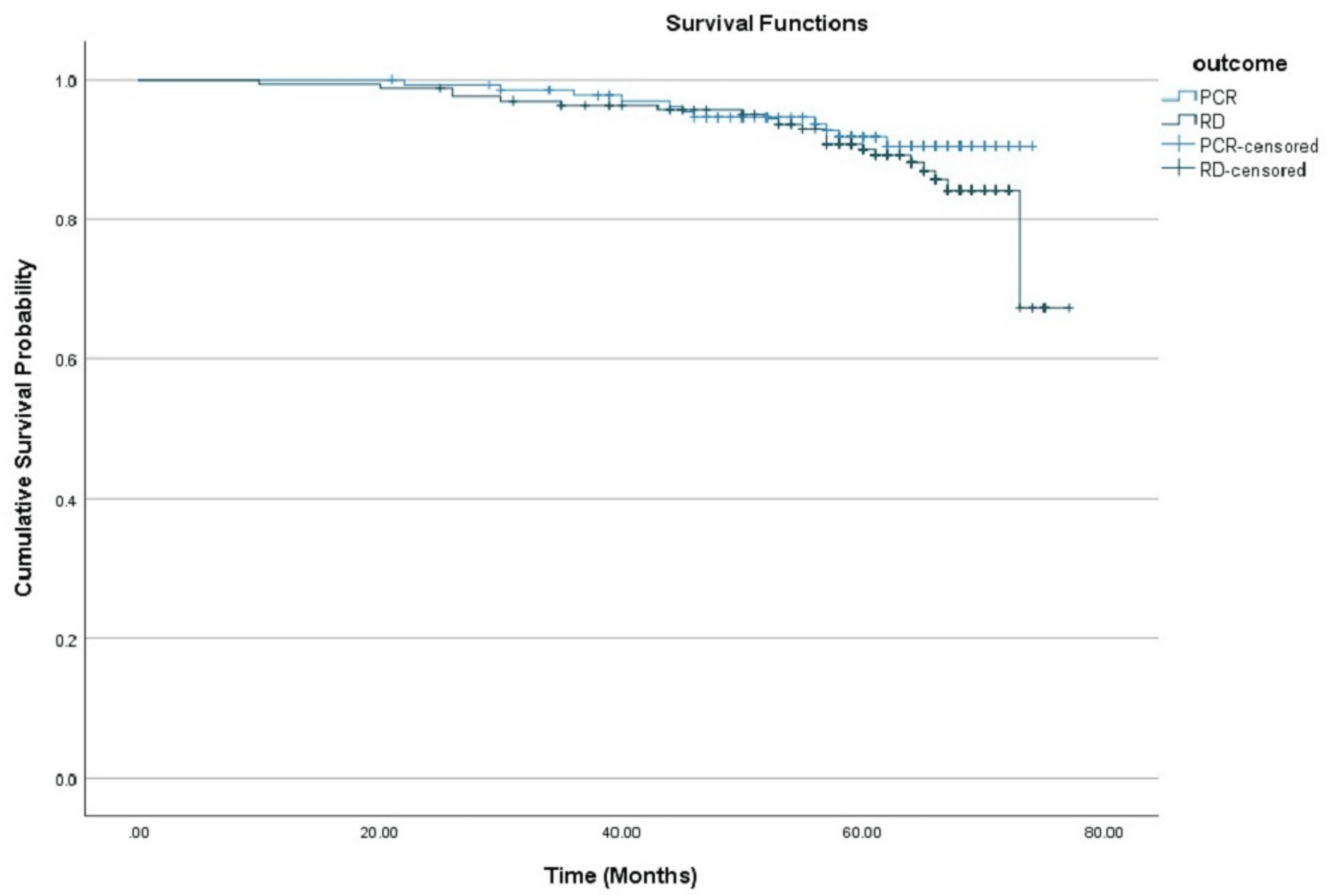
Kaplan-Meier curves comparing overall survival between patients who achieved pCR and those with RD. pCR: Pathological complete response; RD: Residual disease

Among those achieving pCR, the 60-month OS was 91.8% (95% CI: 87.0%-96.8%), compared to 84.1% (95% CI: 77.6%-91.2%) among patients with residual disease, as shown in Figure 6.

Log-rank (Mantel-Cox) tests confirmed statistically significant differences in both PFS (χ²=27.962, df=3, p<0.001) and OS (χ²=23.024, df=2, p<0.001) among the treatment groups, with dual HER2-targeted therapy offering the most favorable long-term outcomes.

## Discussion

BC is a major global health issue and one of the leading causes of mortality worldwide [12]. HER2-positive BC is associated with a poor prognosis and rapid progression. HER2-directed therapies have revolutionized the treatment of BC [13]. However, in low-resource settings, access to these advanced treatments is limited due to various barriers to care [14]. The current retrospective cohort study aimed to assess the outcomes of HER2-directed therapies in patients with non-metastatic HER2-positive BC in a resource-limited setting where these agents are not available for all patients.

The study revealed that HER2-directed therapy is a critical determinant of improved survival in patients with HER2-positive BC. In high-resource settings, the use of pertuzumab and trastuzumab has significantly reduced mortality rates by targeting the HER2 protein [15-17]. In our cohort, 85 (28.3%) patients received dual HER2-directed therapy. These patients demonstrated a high rate of pCR (43.5%). However, the limited access to these therapies in resource-poor settings can result in treatment delays or interruptions, adversely affecting OS.

In a study by Hurvitz et al., a pCR was attained in 44.4% of patients receiving trastuzumab plus pertuzumab, while 55.7% of those treated with docetaxel, carboplatin, and trastuzumab plus pertuzumab achieved a pCR [17]. Another study reported a significantly higher pCR rate in the group receiving pertuzumab plus trastuzumab and chemotherapy compared to the trastuzumab-alone group (45.8% vs. 29.0%, p=0.0141) [18]. Furthermore, disease-free survival rates were 81% for patients treated with trastuzumab plus docetaxel, 84% for those receiving pertuzumab and trastuzumab plus docetaxel, 80% for pertuzumab and trastuzumab, and 75% for pertuzumab and docetaxel [5]. Similarly, the KRISTINE trial reported a pCR rate of 44.4% in the trastuzumab plus pertuzumab group and 55.7% in the docetaxel, carboplatin, and trastuzumab plus pertuzumab group [17]. In contrast, only trastuzumab was administered in the majority of cases in the present cohort due to limited resources.

In the HERA trial, after an 11-year median follow-up, patients who received one year of trastuzumab showed a significant reduction in the risk of disease-free survival events (hazard ratio (HR): 0.76) and mortality (HR: 0.74) compared to those under observation. However, extending trastuzumab therapy to two years did not yield additional survival benefits (HR: 1.02). The 10-year disease-free survival rates were 63% for the observation group, 69% for the one-year trastuzumab group, and 69% for the two-year trastuzumab group [19]. Likewise, a meta-analysis demonstrated that the risk of BC recurrence (relative risk (RR): 0.66, 95% CI: 0.62 to 0.71; p<0.0001) and BC-specific mortality (RR: 0.67, 95% CI: 0.61 to 0.73; p<0.0001) were significantly reduced with trastuzumab plus chemotherapy compared to chemotherapy alone [20]. These findings are consistent with the present study.

Due to resource constraints, approximately one-third of our patients did not receive any anti-HER2-directed treatment (35%), another third received trastuzumab along with chemotherapy (37%), and the remaining third received both trastuzumab and pertuzumab in combination with chemotherapy (27%). Hence, this retrospective analysis provides a valuable real-world comparison of the benefits of HER2 blockade in improving pCR, PFS, and OS.

Our study provides real-world evidence that the addition of trastuzumab, either alone or in combination with pertuzumab, to chemotherapy in HER2-positive early BC significantly improves pCR, as well as PFS and OS outcomes. Importantly, our findings reaffirm that high-grade tumors exhibit greater responsiveness to systemic therapy across the three treatment groups, chemotherapy alone, single-agent HER2 blockade, and dual-agent HER2 blockade, than low-grade tumors, with a corresponding increase in pCR rates. While the addition of HER2-directed therapy markedly improved PFS, a longer duration of follow-up is warranted to determine whether a statistically significant benefit in OS can be observed with dual-agent HER2 blockade versus single-agent therapy.

Despite these favorable findings, several limitations must be acknowledged. Resource limitations constituted a major constraint in this study, as evidenced by the relatively low number of patients receiving dual HER2-directed therapy. Socioeconomic disparities further contributed to delays in treatment initiation and limited access to comprehensive care for many patients, potentially compromising clinical outcomes. Future studies in similar settings are needed to validate these findings and advocate for wider accessibility to HER2-targeted agents in low-resource healthcare environments.

## Conclusions

HER2-directed therapies provide significant benefits in patients with HER2-positive non-metastatic BC. Our findings underscore the need for a comprehensive and integrated approach to BC treatment that includes both HER2-directed agents. Expanding access to these therapies in low-resource settings is critical for improving survival outcomes. Future research should focus on developing practical strategies to address treatment challenges in resource-limited environments, thereby contributing to improved global standards of BC care.
